# Functional consequences of post-COVID-19 syndrome: Evidence from Qatar, 2022

**DOI:** 10.5339/qmj.2026.34

**Published:** 2026-06-15

**Authors:** Nada Adli, Nagah Selim

**Affiliations:** 1 Department of Preventive Medicine, Hamad Medical Corporation (HMC), Doha, Qatar; 2 Community and Preventive Medicine Department, Primary Health Care Corporation (PHCC), Doha, Qatar; 3 Cairo University, Giza, Egypt

**Keywords:** Post-COVID-19 syndrome, physical activity, physical dysfunction, fatigue, dyspnea, Qatar

## Abstract

**Background:**

Despite recovery from acute COVID-19, many individuals experience post-COVID-19 syndrome (PCS), which can impair health and functional status, particularly affecting cardiovascular and pulmonary function in physically active adults.

**Objective:**

To assess the severity of physical dysfunction among patients with PCS in Qatar during 2022.

**Methods:**

An analytic cross-sectional study was conducted among 368 adults with laboratory-confirmed COVID-19 infection between January and July 2022. After providing verbal consent, participants completed structured telephone interviews assessing physical activity, fatigue, and dyspnea. The World Health Organization’s standard case definition for PCS was applied, identifying 159 participants with PCS.

**Results:**

Among the PCS participants, a notable reduction in moderate (13.2%) and vigorous (5.7%) physical activity was observed following COVID-19 infection. Severe fatigue and dyspnea were highly prevalent in PCS participants (45.9% and 37.8%, respectively) and were strongly interrelated. Multivariable analysis showed severe dyspnea independently associated with severe fatigue (odds ratio [OR] = 3.04 [95% CI, 1.46–6.31]), and severe fatigue independently associated with severe dyspnea (OR = 3.11 [95% CI, 1.49–6.49]). No significant associations were observed with sociodemographic characteristics, social support, obesity, chronic disease, or hospitalization.

**Conclusion:**

Fatigue and dyspnea are central, interrelated manifestations of PCS, largely independent of demographic or clinical characteristics. These findings highlight the need for routine screening and the integration of multidisciplinary rehabilitation programs, including pulmonary support and graded physical activity, to address functional impairment in patients with PCS. Longitudinal studies are warranted to clarify the long-term trajectory and underlying mechanisms of PCS.

## 1. INTRODUCTION

A substantial proportion of individuals who recover from acute COVID-19 infection continue to experience persistent symptoms, collectively referred to as post-COVID-19 syndrome (PCS). The World Health Organization (WHO) defines PCS as symptoms lasting 12 weeks or more after acute infection, in the absence of an alternative diagnosis.^1–3^ Global estimates suggest that up to 80% of patients develop at least one long-term symptom that interferes with their ability to resume normal daily activities.^4,5^ These symptoms commonly include fatigue, myalgia, and dyspnea, which can impair physical function, restrict movement and physical activity, and potentially lead to disability.^6^

Fatigue and dyspnea are among the most frequently documented symptoms in individuals with post-acute sequelae of SARS-CoV-2 infection.^4,7^ Studies have shown declines in physical performance and deterioration in functional capacity among COVID-19 survivors after the acute phase. These adverse outcomes are likely multifactorial, resulting from prolonged immobility (e.g., hospitalization or reduced physical activity), physical deconditioning (e.g., muscle atrophy and reduced strength due to inactivity), and the direct pathophysiological effects of SARS-CoV-2, including systemic inflammation and multi-organ involvement.^8,9^

Dyspnea has been frequently reported as a symptom across 26 PCS studies, with a median frequency ranging from 27.6% to 50.0%.^10,11^ Additionally, fatigue emerged as the most common PCS symptom reported worldwide, irrespective of the severity of the initial COVID-19 infection and the hospitalization status during the acute illness. The prevalence varies widely, ranging from 28% to 87% across different studies,^12–14^ and in a comprehensive analysis of 25 studies, participants frequently experienced fatigue, with a median frequency ranging from 31.0% to 57.0%.^15^

Moreover, physical activity was negatively impacted during and after COVID-19 infection. A study conducted in the Netherlands and Flanders involving 239 COVID-19 patients assessed exercise patterns before and 6 months after symptom onset.^16^ Walking time decreased to 3 months post-infection (60 minutes/week vs. 120 minutes/week pre-COVID-19; P < 0.05). Although walking time improved by 6 months (90 minutes/week), it remained significantly lower than pre-infection levels.^16^ Another study of 156 patients found that individuals were less likely to meet the recommended 150 minutes of physical activity per week after COVID-19 compared to pre-infection.^17^ The reason behind that was investigated by Pasini et al. who reported that individuals with new-onset fatigue, muscle weakness, and dyspnea after COVID-19, despite being asymptomatic beforehand, exhibited elevated serum ferritin, C-reactive protein, and lactate dehydrogenase (LDH), along with reduced albumin and abnormal coagulation parameters.^18^ These results suggest that systemic inflammation and altered cellular processes may contribute to persistent symptoms such as fatigue and musculoskeletal pain.^18^

Despite the growing global evidence, a significant research gap remains in the Middle East and Gulf region regarding the persistence of PCS symptoms and their long-term impact on physical functioning. This study aimed to address this gap by identifying the prevalence and severity of physical dysfunction associated with PCS in Qatar and by examining its relationship with socio-demographic and health-related characteristics among adults.

## 2. MATERIALS AND METHODS

This cross-sectional study, part of a thesis project,^19^ analyzed confirmed COVID-19 cases in Qatar from January to July 2022. Data were obtained from the Primary Health Care Corporation’s (PHCC) Business Intelligence Unit (BIU), which oversees electronic records across 31 centers serving over 1.6 million people and supports national COVID-19 reporting.^20^

This study was conducted as part of a larger project^19^ that initially enrolled 368 patients with laboratory-confirmed COVID-19 infection (PCR or rapid antigen test) in Qatar between January and July 2022, identified through the BIU of the PHCC.

Eligible participants were adults aged ≥18 years or older who were able to communicate in Arabic or English, with no restrictions on sex or nationality. Individuals with pre-existing physical functional impairment were excluded. Participants were selected using computer-generated random numbers and contacted by telephone. Those who provided consent verbally completed structured interviews of approximately 30 minutes’ duration.

Participants with PCS were identified according to the WHO’s standard case definition, totaling 159 individuals. The remaining 209 participants were classified as the non-PCS group.

The outcome of this study aimed to measure the severity of physical dysfunction, specifically fatigue, dyspnea, and physical activity levels, among post-COVID-19 patients.

Fatigue was assessed using the Fatigue Severity Scale (FSS),^21–23^ a validated and reliable instrument with a Cronbach’s alpha of 0.928. The FSS comprised nine items rated on a 7-point Likert scale, ranging from 1 (“Disagree”) to 7 (“Agree”). A total score of less than 36 suggested that participants were unlikely to experience clinically significant fatigue, whereas a score of 36 or higher indicated severe fatigue. Accordingly, participants were categorized into two groups: severe fatigue (FSS ≥ 36) and non-severe fatigue (FSS < 36).

Dyspnea was evaluated using the Medical Research Council (MRC) Breathlessness Scale,^24^ a validated tool consisting of a single question with five graded statements. Each statement was assigned a grade from 1 to 5, where grade 1 corresponded to “I only get breathless with strenuous exercise,” and grade 5 corresponded to “I am too breathless to leave the house, or I am breathless when dressing or undressing”. Based on these grades, dyspnea was classified into three categories: mild (grade 1), moderate (grades 2–3), and severe (grades 4–5).

Furthermore, physical activity was also assessed by evaluating the frequency of moderate- and vigorous-intensity exercises before and after COVID-19 infection.^16,25,26^ Participants reported their activity levels using the following categories: “Weekly,” “Some weeks,” “Most weeks,” and “Never.”^17^ Differences in physical activity levels before and after infection were calculated and categorized into three groups: no change in physical activity, increase in physical activity, and decrease in physical activity.

Independent variables included sociodemographic and economic characteristics: age, gender, nationality, marital status, education, employment, perceived monthly income, and social support, which was assessed using the Oslo Social Support Scale (OSSS-3), a validated three-item instrument measuring the number of close confidants, perceived concern from others, and access to practical help from neighbors. Total scores range from 3 to 14, with higher scores indicating greater social support. Scores were categorized as poor (3–8), moderate (9–11), or strong (12–14) social support.^27^ In addition, health-related variables included the presence and type of comorbidities (e.g., hypertension, diabetes, asthma), BMI (obese or non-obese), and history of hospital admission.

This study was approved by the PHCC Ethical Committee (PHCC-IRB, protocol number: PHCC/DCR/2022/06/31). Verbal consent was obtained, and the research followed the Declaration of Helsinki and Good Clinical Practice guidelines.

Data were analyzed using SPSS v25. Descriptive statistics summarized participant characteristics, and normality was assessed with the Kolmogorov-Smirnov test. Chi-squared tests examined associations with post-COVID-19 physical dysfunction, and logistic regression identified independent predictors, reporting adjusted odds ratios (ORs) with 95% CIs. A P-value <0.05 was considered significant.

## 3. RESULTS

Table 1 shows changes in physical activity before and after COVID-19 infection. Among PCS participants (*n* = 159), weekly moderate-intensity activity declined from 27.7% to 20.1%, while vigorous activity decreased from 13.2% to 8.2%. In the non-PCS group, moderate activity fell from 23.9% to 18.7% and vigorous activity from 10.0% to 9.1%. However, none of these associations were statistically significant.

**Figure 1.**
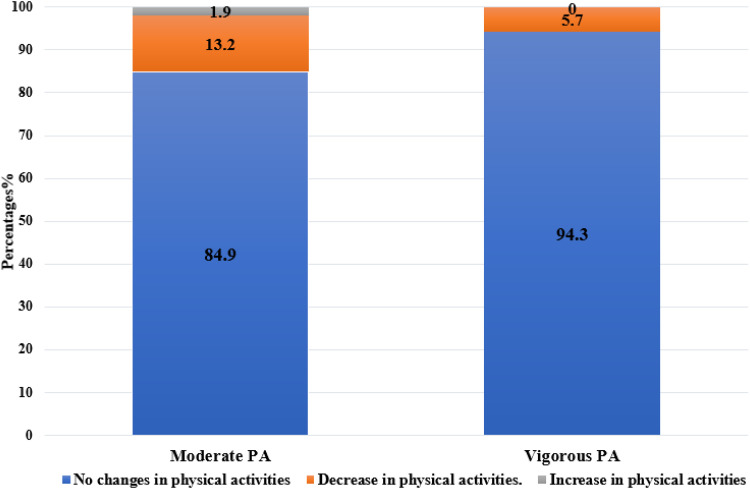
Change of moderate and vigorous physical activities among PCS cases before and after COVID-19 infection in Qatar during 2022 (*n* = 159).

Regarding physical activities, Figure 1 illustrates alterations in moderate and vigorous physical activities before and after the onset of COVID-19 infection.

It was noteworthy that most individuals who suffered from PCS did not experience any significant changes in their physical activity levels.

However, within the PCS group, a notable reduction of 13.2% in moderate physical activities and a 5.7% decline in vigorous physical activities were observed.

Conversely, we observed a marginal increase in moderate physical activities (1.9%) after the COVID-19 infection among individuals with PCS. However, no significant increase in vigorous physical activities was observed in the PCS group after the COVID-19 infection.

Among all study participants, the majority reported mild dyspnea following COVID-19 infection, accounting for 81.0% (*n* = 298) of the sample. In contrast, severe dyspnea was reported by only 2.5% of participants during 2022, as presented in Table 2.

**Figure 2.**
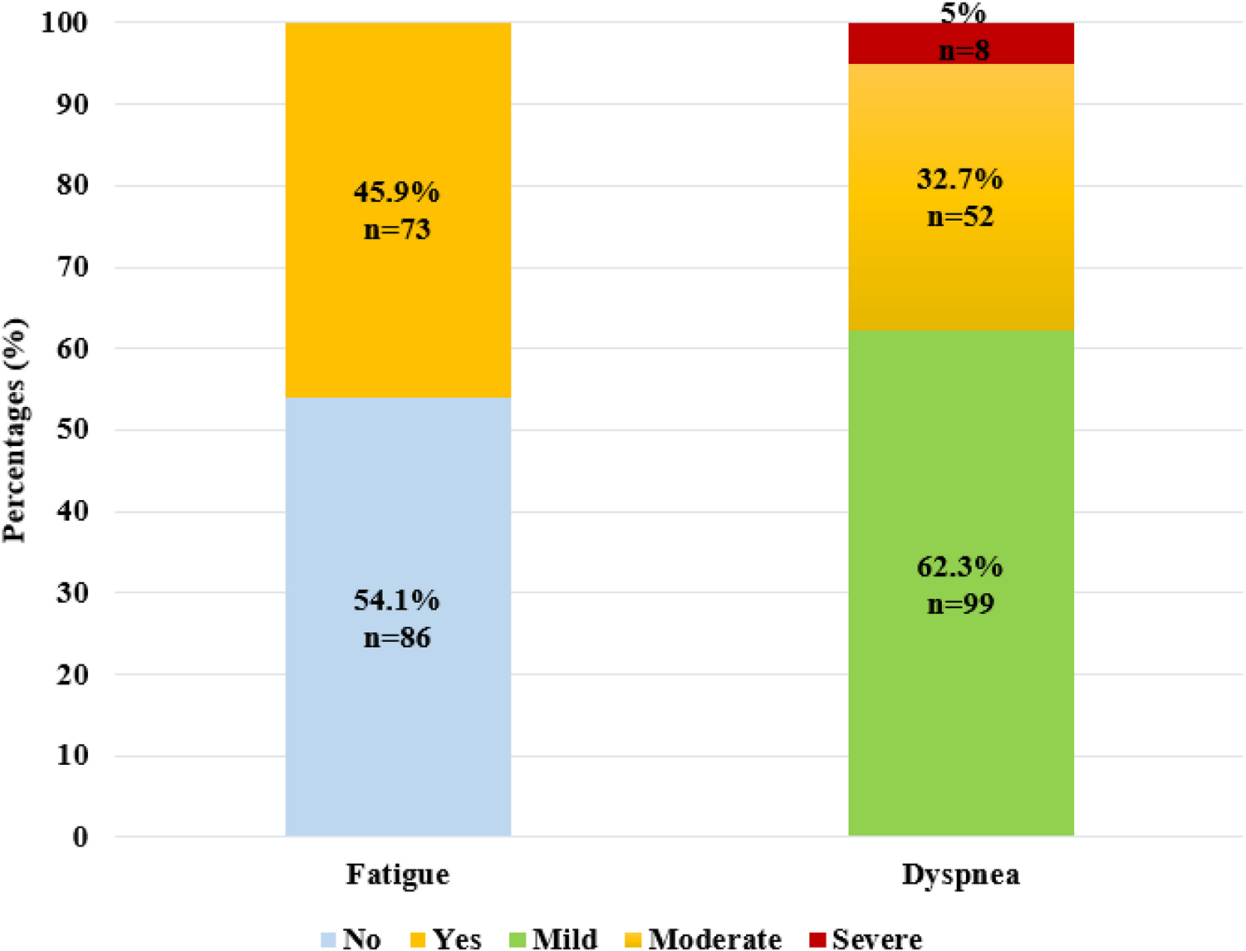
Frequency distribution of the severity level of physical dysfunction among adults with post-COVID-19 syndrome in Qatar during 2022 (*n* = 159).

Figure 2 explores the severity level of physical impairment among PCS cases. Severe fatigue was reported among 45.9% (*n* = 73) of PCS participants, with a mean score of 30.8 ± 16.1 (95% CI, 15–45). About one-third of the participants (32.7%; *n* = 52) had moderate dyspnea, while (5%; *n* = 8) reported a severe level of dyspnea.

Applying the bivariate analysis, we found that dyspnea and severe fatigue had a strong and statistically significant association with PCS.

Severe fatigue was more prevalent among the PCS group compared to the non-PCS group (45.9% vs. 11.5%; *P* = 0.000), with a statistically significant difference in the mean score between both groups (30.8 ± 16.1 vs. 14.5 ± 10.8) and a P-value < 0.001.

Additionally, dyspnea was more frequently observed among PCS patients than in people without PCS (37.75% vs. 4.8%), with P-value < 0.001.

In contrast, there was no statistically significant difference between PCS and non-PCS groups regarding changes in moderate and vigorous physical activities before and after COVID-19 infection, despite an observational difference in the decline of moderate and vigorous physical activities between both PCS group and those without PCS group pre- and post-COVID-19 infection (13.2% vs. 8.6%) and (5.7% vs. 2.9%), respectively, as shown in Table 3.

Table 4 shows the association between sociodemographic and health-related factors and physical dysfunction among PCS participants. Most characteristics, including age, gender, nationality, marital status, education, employment, income, social support, obesity, chronic disease history, and hospitalization, were not significantly associated with severe fatigue or dyspnea. Females were more represented among those with severe fatigue, but the difference was not statistically significant (*P* = 0.24). Severe fatigue and severe dyspnea were strongly interrelated, with participants reporting fatigue more likely to experience dyspnea (50.7% vs. 26.7%; *P* = 0.002), highlighting the coexistence of these persistent PCS symptoms.

Multivariable logistic regression identified a strong bidirectional relationship between severe fatigue and severe dyspnea among PCS patients. Severe dyspnea independently associated with severe fatigue (OR = 3.04 [95% CI, 1.46–6.31]; *P* = 0.003), and severe fatigue independently associated with severe dyspnea (OR = 3.11 [95% CI, 1.49–6.49]; *P* = 0.002). No sociodemographic or clinical factors, including age, gender, social support, chronic disease, or hospitalization, were significant predictors of either symptom as shown in Table 5.

## 4. DISCUSSION

This study aimed to assess the severity of physical dysfunction among patients with PCS in Qatar during 2022. Individuals infected with SARS-CoV-2 may continue to experience persistent symptoms well beyond recovery from the acute phase of illness. These ongoing manifestations, collectively referred to as PCS, vary in type, severity, and duration.^28^ Although some patients report gradual improvement over time, a substantial proportion continue to suffer from long-lasting symptoms that interfere with daily functioning and hinder return to normal life. These symptoms, including fatigue, myalgia, and dyspnea, can significantly impair physical function, limit mobility and physical activity, and daily routine.^17^

Numerous studies have demonstrated a strong association between PCS and impaired physical function. For example, Belli et al. reported that approximately half of Italian post-COVID-19 patients experienced severe limitations in physical function and activities of daily living following hospital discharge.^29^ Similarly, Lemhöfer et al. found that 49% of post-COVID-19 patients in Germany reported activity limitations and participation restrictions.^30^ Jacobson et al. further demonstrated that 46% of mildly affected individuals and 73% of hospitalized patients experienced impairments in work and daily activities 3 to 4 months after the initial infection.^31^ In addition, a scoping review of 34 studies reported fatigue as the most prevalent physical symptom following COVID-19, with prevalence ranging from 28% to 87%, alongside reduced physical capacity, diminished role functioning, and limitations in usual care and daily activities in 15% to 54% of patients.^32^

In the present study, we assessed physical function impairment in terms of physical activity, dyspnea, and fatigue. A significant reduction in both moderate (13.2%) and vigorous (5.7%) physical activity levels was observed among individuals with PCS following COVID-19 infection. These findings are consistent with previous reports indicating that decreased physical activity is a common sequela of COVID-19.^17,29–32^ Moreover, the observed decline in physical function and activity levels aligns with evidence from earlier studies and systematic reviews documenting long-term functional impairments among survivors of previous coronavirus infections, including SARS and MERS.^29,33^ Notably, one systematic review reported lung function abnormalities and impaired diffusing capacity for carbon monoxide among SARS and MERS survivors, with a pooled prevalence of 27% (95% CI, 15%–45%),^33^ highlighting the potential long-term pulmonary sequelae of coronavirus infections.

Dyspnea was significantly more prevalent among PCS patients in our cohort compared with those without PCS. More than one-third of PCS patients (37.7%) reported moderate to severe dyspnea based on the MRC dyspnea scale, whereas only 4.8% of non-PCS individuals experienced similar severity. This finding is comparable to the results reported by Tabacof et al. who observed moderate to severe dyspnea in approximately 40% of PCS patients.^17^ Nevertheless, other studies have documented lower prevalence rates of dyspnea, ranging between 25% and 29%, using the MRC dyspnea scale,^34,35^ and other tools such as Grewal et al.^36^ and Nugent et al.^37^ studies. Such variability may be explained by differences in follow-up duration, study population characteristics, symptom assessment tools, and the severity of the acute COVID-19 infection.

The underlying mechanisms of post-COVID-19 dyspnea remain a subject of ongoing debate. Several studies have attributed persistent dyspnea to post-infectious respiratory changes, including alterations in lung parenchyma, interstitial involvement, and pulmonary fibrosis, which may lead to reduced lung capacity and impaired pulmonary function.^38,39^ Other investigations have emphasized the role of reduced physical exercise capacity and deconditioning as key contributors to persistent dyspnea.^40,41^ Given the multifactorial nature of post-COVID-19 dyspnea, further longitudinal and mechanistic studies are required to clarify its pathophysiology and contributing factors.

Fatigue represents another hallmark symptom of PCS and is among the most frequently reported long-term consequences of COVID-19, with prevalence estimates ranging from 28% to 87% globally.^12–14^ In our study, fatigue was significantly more prevalent among PCS patients compared with those without PCS (45.9% vs. 11.5%; *P* < 0.001), with a median FSS score of 33.0 (interquartile range, 15–45). These findings are consistent with observations from previous studies, including those by Tabacof et al. Venturelli et al. Beyer et al. and Menges et al. all of which reported a high burden of fatigue among post-COVID-19 patients.^17,34,35,42^ However, the prevalence of fatigue in our cohort was lower than that reported by Tabacof et al.^17^ (78%)and higher than that observed by Menges et al.^35^ (55%), likely reflecting differences in study design, assessment tools, and clinical characteristics of the study populations.

Importantly, our findings demonstrate a strong and independent association between severe fatigue and severe dyspnea. Multivariable analysis revealed that patients with severe fatigue had more than a threefold increased likelihood of experiencing severe dyspnea, and vice versa. This bidirectional relationship suggests shared or overlapping pathophysiological mechanisms such as impaired oxygen utilization, autonomic dysfunction, deconditioning, or persistent inflammatory responses and underscores the need for integrated, symptom-focused management approaches.^43^ Addressing fatigue and dyspnea concurrently may therefore be essential for improving physical function and overall quality of life among individuals with PCS.

Although most sociodemographic factors and hospital admission status were not statistically significant in our study, previous literature from different settings, including the Middle East, has reported that these variables may still play an important role in post-COVID-19 outcomes, particularly fatigue, dyspnea, and reduced physical function. For instance, in 2021, a large United Kingdom cohort study by Huang et al. included 1733 hospitalized COVID-19 survivors, demonstrating that older age and female sex were associated with persistent fatigue and dyspnea up to 6 months after infection.^44^ Similarly, a study conducted by Sudre et al. in 2021, among 4182 participants, found that demographic factors such as age, sex, and comorbidities were significant predictors of long COVID symptoms, including breathlessness and functional limitation.^45^ In Italy, during 2020, Carfì et al. reported that a high proportion of previously hospitalized patients experienced persistent fatigue and dyspnea after recovery,^46^ while Evans et al. further highlighted that hospitalization during acute infection was associated with more severe long-term functional impairment.^47^

Evidence from the Middle East also supports similar patterns. In Saudi Arabia, a study by Mahmoud et al.^47^ found that female gender and comorbidities were associated with higher odds of persistent fatigue and reduced functional capacity following COVID-19 infection. In Qatar, a study by Adli et al. described a persistent symptom burden, including fatigue and breathlessness, particularly among those with more severe acute illness, although demographic associations were less consistent across outcomes.^19^

This study provides valuable insights into PCS in Qatar, using a standardized case definition and validated tools to assess fatigue, dyspnea, and physical activity. Random sampling across diverse participants enhances the representativeness of the findings, and structured telephone interviews enabled safe and feasible data collection. Moreover, we had well-trained data collectors who contributed to the data quality and accuracy, enhancing our findings’ overall reliability.

This study has several limitations. The cross-sectional design limits causal inference and temporal interpretation. Additionally, reliance on self-reported data without objective measures, such as pulmonary function or exercise testing, may introduce reporting bias and limit the strength of the findings. The telephone-based recruitment approach may also have introduced selection bias by underrepresenting individuals with severe symptoms, cognitive impairment, or communication barriers, potentially affecting external validity. Furthermore, physical activity was assessed using a non-standardized tool adopted from a previously published study and evaluated through face validity only, which may limit measurement precision and result in some loss of quantitative information. These factors may affect the generalizability of the findings.

## 5. CONCLUSION

This study found that moderate- and vigorous-intensity physical activity declined after COVID-19 infection, but these changes were not independently linked to PCS. Severe fatigue and dyspnea were highly prevalent among PCS participants and strongly interrelated, with each symptom independently predicting the other. No significant associations were observed with sociodemographic or clinical factors, indicating that persistent fatigue and dyspnea are central, interrelated features of PCS, largely independent of background characteristics.

Routine screening for fatigue and dyspnea should be included in post-COVID-19 care, alongside multidisciplinary reehabilitation with pulmonary support and graded physical activity. Longitudinal studies are needed to clarify the long-term course and mechanisms of these symptoms.

## ACKNOWLEDGEMENT

The authors would like to sincerely thank the Qatar Medical Journal for its support in facilitating the publication process.

## FUNDING

This research did not receive any specific grant from funding agencies in the public, commercial, or not-for-profit sectors.

## CONFLICT OF INTEREST

The authors declare that the research was conducted in the absence of any commercial or financial relationships that could be construed as potential conflicts of interest.

## ETHICAL APPROVAL

This study was approved by the Primary Health Care Corporation Ethical Committee (PHCC-IRB) under protocol number: PHCC/DCR/2022/06/31. Verbal consent was obtained from each participant before the interview. The study was conducted in full conformance with the principles of the Declaration of Helsinki and Good Clinical Practice.

## AUTHOR CONTRIBUTIONS

NA: Conceptualization, methodology, investigation, formal analysis, data curation, project administration, writing – original draft, and writing – review and editing. NS: Methodology, formal analysis, data curation, supervision, review, and editing.

## DATA AVAILABILITY STATEMENT

The data sets generated during and/or analyzed during the current study are available from the corresponding author on reasonable request.

## DISCLOSURE OF AI USE

The authors also declare that artificial intelligence (AI) tools were used solely for language editing and formatting of the manuscript. No AI tools were used for data analysis, interpretation of results, or generation of scientific content, and all intellectual content and conclusions are entirely the responsibility of the authors.

## Figures and Tables

**Table 1.** Frequency distribution of physical activities before and after COVID-19 infection among the study participants and their association with post-COVID-19 syndrome in Qatar during 2022 (*N* = 368).

	All study participants				
	Post-COVID-19 syndrome (*n* = 159)		Not post-COVID-19 syndrome (*n* = 209)		
Variables	*n*	%	*n*	%	*P*-value
**Regular moderate-intense exercise BEFORE COVID-19 infection (150 minutes per week, e.g., walking, dancing, and playing doubles tennis, or raking the yard, slow swimming)**					
Weekly	44	(27.7)	50	(23.9)	0.462
Some weeks	21	(13.2)	27	(12.9)	
Most weeks	17	(10.7)	34	(16.3)	
Never	77	(48.4)	98	(46.9)	
**Regular moderate-intense exercise AFTER COVID-19 infection (150 minutes per week, e.g., walking, dancing, and playing doubles tennis, or raking the yard, slow swimming)**					
Weekly	32	(20.1)	39	(18.7)	0.377
Some weeks	24	(15.1)	31	(14.8)	
Most weeks	14	(8.8)	31	(14.8)	
Never	89	(56.0)	108	(51.7)	
**Regular vigorous intensity exercise BEFORE COVID-19 infection (150 minutes per week, e.g., jogging, running, fast cycling, fast swimming, and walking briskly up a hill, participating in a strenuous fitness class)**					
Weekly	21	(13.2)	21	(10.0)	0.163
Some weeks	2	(1.3)	7	(3.3)	
Most weeks	0	(0.0)	4	(1.9)	
Never	136	(85.5)	177	(84.7)	
**Regular vigorous intensity exercise AFTER COVID-19 infection (150 minutes per week, e.g., jogging, running, fast cycling, fast swimming, and walking briskly up a hill, participating in a strenuous fitness class)**					
Weekly	13	(8.2)	19	(9.1)	0.986
Some weeks	3	(1.9)	5	(2.4)	
Most weeks	2	(1.3)	3	(1.4)	
Never	141	(88.7)	182	(87.1)	

**Table 2.** The frequency distribution of dyspnea among adults infected with COVID-19 in Qatar during 2022 (*N* = 368).

Medical Research Council Dyspnea scale	Frequency (*n*)	Percentage (%)
**Grade 1:** I only get breathless with strenuous exercise.	298	(81.0)
**Grade 2:** I get short of breath when hurrying on the level or walking up a slight hill.	45	(12.2)
**Grade 3:** I walk slower than people of the same age on the level because of breathlessness, or I have to stop for breath when walking at my own pace on the level.	16	(4.3)
**Grade 4:** I stop for breath after walking about 100 m or after a few minutes on the level.	8	(2.2)
**Grade 5:** I am too breathless to leave the house, or I am breathless when dressing or undressing.	1	(0.3)

**Table 3.** The association between post-COVID-19 syndrome and physical dysfunction among adults in Qatar during 2022 (*N* = 368).

	Group post COVID-19 Syndrome				
	Yes (*n* = 159)		No (*n* = 209)		
Variables	*n*	(%)	*n*	(%)	*P*-value
**Changes in moderate physical activities before and after COVID-19 infection**					
No changes in physical activities	135	(84.9)	185	(88.5)	0.312
Decrease in physical activities	21	(13.2)	18	(8.6)	
Increase in physical activities	3	(1.9)	6	(2.9)	
**Changes in vigorous physical activities before and after COVID-19 infection**					
No changes in physical activities	150	(94.3)	201	(96.2)	0.197
Decrease in physical activities.	9	(5.7)	6	(2.9)	
Increase in physical activities.	0	(0.0)	2	(1.0)	
**Severe fatigue**					
Yes	73	(45.9)	24	(11.5)	0.000***
No	86	(54.1)	185	(88.5)	
**Fatigue Severity Median score (IQR)**	**33.0 [15–45]**		**12.0 [9–36]**		0.000***
**Severe dyspnea**					
Yes	60	(37.7)	10	(4.8)	0.000***
No	99	(62.3)	199	(95.2)	

*P < 0.05; **P < 0.01, ***P < 0.001. Bold values indicate statistically significant results.

**Table 4.** The association between the sociodemographic and health-related characteristics and the severity of physical dysfunction among post-COVID-19 patients in Qatar during 2022 (*n* = 159).

	Post-COVID-19 syndrome cases (*n* = 159)									
	Severe fatigue					Severe dyspnea				
Variable	Yes (*n* = 73)		No (*n* = 86)		*P*-value	Yes (*n* = 60)		No (*n* = 99)		*P*-value
	*n*	(%)	*n*	(%)		*n*	(%)	*n*	(%)	
**Age**					0.626					0.352
18–24	3	(4.1)	7	(8.1)		3	(5.0)	7	(7.1)	
25–39	49	(67.1)	55	(64.0)		36	(60.0)	68	(68.7)	
40 and above	21	(28.8)	24	(27.9)		21	(35.0)	24	(24.2)	
**Gender**					0.244					0.662
Male	20	(27.4)	31	(36.0)		18	(30.0)	33	(33.3)	
Female	53	(72.9)	55	(64.0)		42	(70.0)	66	(66.7)	
**Nationality**					0.077					0.573
Qatari	4	(5.5)	12	(14.0)		5	(8.3)	11	(11.1)	
Non-Qatari	69	(94.5)	74	(86.0)		55	(91.7)	88	(88.9)	
**Marital status**					0.535					0.753
Not married	21	(28.8)	21	(24.4)		15	(25.0)	27	(27.3)	
Married	52	(71.2)	65	(75.6)		45	(75.0)	72	(72.7)	
**Education status**					0.647					0.599
Up to secondary education	13	(17.8)	13	(15.1)		11	(42.3)	15	(57.7)	
**University/higher education**	60	(82.2)	73	(84.9)		49	(36.8)	84	(63.2)	
**Employment status**					0.257					0.430
Employed	53	(72.6)	69	(80.2)		44	(73.3)	78	(78.8)	
Not employed	20	(27.4)	17	(19.8)		16	(26.7)	21	(21.2)	
**Perceived monthly income**					0.669					0.900
Not enough at all	28	(38.4)	36	(41.9)		24	(40.0)	40	(40.4)	
Enough	39	(53.4)	46	(53.5)		33	(55.0)	52	(52.5)	
More than enough	6	(8.2)	4	(4.7)		3	(5.0)	7	(7.1)	
**Social Support Scale**					0.233					0.111
Poor social support	63	(86.3)	68	(79.1)		30	(50.0)	34	(34.3)	
Moderate social support	10	(13.7)	18	(20.9)		21	(35.0)	40	(40.4)	
Strong social support	0	0	0	0		9	(15.0)	25	(25.3)	
**Obese**					0.282					0.302
Yes	58	(79.5)	62	(72.1)		48	(80.0)	72	(72.7)	
No	15	(20.5)	24	(27.9)		12	(20.0)	27	(27.3)	
**History of chronic diseases**					0.496					0.082
Yes	24	(32.9)	24	(27.9)		23	(38.3)	25	(25.3)	
No	49	(67.1)	62	(72.1)		37	(61.7)	74	(74.7)	
**Need hospital admission**					0.509					0.672
Yes	3	(4.1)	6	(7.0)		4	(6.7)	5	(5.1)	
No	70	(95.9)	80	(93.0)		56	(93.3)	94	(94.9)	
**Required ICU admission Among hospitalized patients**					1.000					0.368
Yes	0	(0.0)	1	(16.7)		1	(25.0)	1	(20.0)	
No	3	(100)	5	(83.3)		3	(75.0)	4	(80.0)	
**Vaccination status**					0.515					0.007**
Yes	67	(91.8)	82	(95.3)		52	(86.7)	97	(98.0)	
No	6	(8.2)	4	(4.7)		8	(13.3)	2	(2.0)	
**Severe fatigue**										0.002**
Yes						37	(61.7)	36	(36.4)	
No						23	(38.3)	63	(63.6)	
**Severe dyspnea**					0.002*					
Yes	37	(50.7)	23	(26.7)						
No	36	(49.3)	63	(73.3)						

*P < 0.05; **P < 0.01, ***P < 0.001. Bold values indicate statistically significant results.

**Table 5.** Logistic regression between the sociodemographic and health related characteristics and the severity of physical dysfunction among post-COVID-19 patients in Qatar during 2022 (*n* = 159).

	Severe fatigue							Severe dyspnea						
	B	SE	Wald	Significance	Exp (B)	95% CI for Exp (B)		B	SE	Wald	Significance	Exp (B)	95% CI for Exp (B)	
Constant	−0.16	0.159	1.061	0.303	0.849	Lower	Upper	−0.50	0.164	9.369	0.002	0.606	Lower	Upper
**Age**														
18–24														
25–39	1.084	0.852	1.617	0.204	2.956	0.556	15.710	−0.21	0.853	0.061	0.805	0.810	0.152	4.307
40 and above	0.860	0.895	0.923	0.337	2.363	0.409	13.648	0.279	0.888	0.099	0.754	1.322	0.232	7.540
**Gender**														
Male														
Female	0.417	0.385	1.177	0.278	1.518	0.714	3.227	0.163	0.396	0.170	0.680	1.177	0.542	2.560
**Nationality**														
Qatari														
Non-Qatari	1.056	0.661	2.550	0.110	2.875	0.786	10.507	0.088	0.633	0.019	0.890	1.092	0.316	3.777
**Marital status**														
Not married														
Married	−0.57	0.443	1.667	0.197	0.564	0.237	1.345	−0.01	0.466	0.001	0.972	0.984	0.395	2.452
**Education status**														
Up to secondary education														
University/higher education	−0.48	0.491	0.980	0.322	0.615	0.235	1.610	0.007	0.509	0.000	0.988	1.007	0.372	2.730
**Perceived monthly income**														
Not enough at all														
Enough	0.040	0.365	0.012	0.914	1.040	0.508	2.129	0.133	0.389	0.118	0.731	1.143	0.534	2.447
More than enough	0.672	0.800	0.706	0.401	1.959	0.408	9.402	0.080	0.828	0.009	0.923	1.083	0.214	5.493
**Social support scale**														
Strong social support														
Poor social support	−0.28	0.407	0.497	0.481	0.750	0.338	1.667	−0.38	0.412	0.871	0.351	0.681	0.303	1.527
Moderate social support	0.489	0.488	1.003	0.317	1.630	0.626	4.245	−1.14	0.551	4.325	0.038	0.318	0.108	0.936
**History of chronic diseases**														
No [Ref]														
Yes	0.208	0.400	0.270	0.603	1.231	0.562	2.693	0.617	0.412	2.237	0.135	1.853	0.826	4.160
**Need hospital admission**														
No [Ref]														
Yes	−0.60	0.779	0.608	0.436	0.545	0.118	2.508	0.724	0.778	0.865	0.352	2.062	0.449	9.471
**Severe fatigue**														
No [Ref]														
Yes								1.135	0.375	9.142	0.002*	3.110	1.490	6.488
**Severe dyspnea**														
No [Ref]														
Yes	1.112	0.373	8.898	0.003*	3.040	1.464	6.313							

*P < 0.05; **P < 0.01, ***P < 0.001. Bold values indicate statistically significant results.
